# Mismatches in Suppliers’ and Demanders’ Cognition, Willingness and Behavior with Respect to Ecological Protection of Cultivated Land: Evidence from Caidian District, Wuhan, China

**DOI:** 10.3390/ijerph17041156

**Published:** 2020-02-12

**Authors:** Siyu Zhang, Weiyan Hu, Jiaojiao Zhang, Mengran Li, Qingying Zhu

**Affiliations:** College of Public Administration, Huazhong Agricultural University, Wuhan 430070, China; zhangsy@webmail.hzau.edu.cn (S.Z.); JiaojiaoZhang@webmail.hzau.edu.cn (J.Z.); MengranLi@webmail.hzau.edu.cn (M.L.); qingyingzhu@webmail.hzau.edu.cn (Q.Z.)

**Keywords:** ecological protection of cultivated land, the theory of planned behavior, suppliers and demanders, structural equation model

## Abstract

Cultivated land systems have an enormous ecological function value with respect to water conversation, nutrient circulation and climate regulation. The people’s cognition, willingness and behavior may prove to be pivotal in ecologically protecting cultivated land. The purpose of this paper is to explore suppliers’ and demanders’ cognition, willingness and behavior with respect to the ecological protection of cultivated land. The second-order structural equation model was employed, and a five-point Likert scale was designed. Based on data obtained from a questionnaire surveyed on a sample of 460 (farming and no-farming households) from Caidian district, Wuhan, China, the results show that within/between suppliers and demanders, there are mismatches in willingness as well as behavior to ecologically protect cultivated land. In the suppliers group and in the demanders group, there are mismatches with respect to the interactive mechanism of cognition, willingness and behavior in the ecological protection of cultivated land. Three factors, subjective norms, attitude toward behavior, and perceived behavior control, affect willingness and behavior with respect to protection differently between the suppliers and the demanders. The “intermediary” effects of willingness and behavior in the ecological protection of cultivated land only appear in the demanders model, and are not significant in the suppliers model. In addition, another finding was that farmers’ behavior as suppliers and demanders of ecological protection of cultivated land were mismatched. The article contributes firstly to exploring the ecological protection of cultivated land from the perspective of suppliers’ and demanders’ subjective psychology. Farmers with “dual roles” as suppliers and demanders should pay more attention to the ecological protection of cultivated land. Their cognition and skills in the ecological protection of cultivated land are in need of improvement. It is also necessary to bridge the gap between suppliers and demanders; access to the knowledge of the ecological value of cultivated land will incentivize their behavior with respect to the ecological protection of cultivated land.

## 1. Introduction

Cultivated land is the material basis for human survival and development, having also important functions in production, ecology, society and landscape culture [1,2,3]. The ecological function of cultivated land is to protect ecological diversity, conserve water sources, recycle water sources, and regulate climate [4]. Nowadays, the problems of the ecological security of cultivated land and the protection of cultivated land resources are severe, especially in China, where cultivated land represents less than 10% of the world´s cultivated land area, but the amount of fertilizer applied is close to 1/3 of the world’s total [5]. The China Geological Survey’s “Report on China’s Cultivated Land Geochemical Survey” shows that the medium–heavy polluted area of heavy metals reached 2.33 million hectares, and the light–slightly polluted area also had nearly 5.27 million hectares from 2005 to 2014 [6].

Responding to this situation, the Chinese government has formulated several policies to encourage the development of new agricultural forms, like circular agriculture, ecological agriculture, and farming to show the importance of the ecological protection of cultivated land. In 2013, China’s Land and Resources Ministry (MLRC) issued the *Notice on improving the level of cultivated land protection and Comprehensively Strengthening the construction and management of cultivated land quality*, which remarks that the cultivated land protection policy has officially entered the stage of the protection and management of cultivated land in quantity, quality and ecology. In January 2017, the CPC Central Committee and the State Council released and implemented the *Guidance on Strengthening the Protection and Improving the Balance of Requisition and Compensation of Cultivated Land*. Those policies informed that people’s scientific knowledge of the functions and current situation of cultivated land is the key to protect it. People’s weak awareness, especially farmers’ weak cognition of the ecological function of cultivated land, and the limited access to information have a negative impact on protection [7]. People’s intention and behavior plays a crucial role in the ecological protection of cultivated land [8,9,10]. Since 2013, the government has formulated a series of policies to support the ecological protection of cultivated land; however, the supply of ecological function of cultivated land is continuously declining. There are mismatches between the supply and the increasing demand for ecological function of cultivated land, which restrict the sustainable development of agricultural and rural areas in China [11].

Existing research has paid more attention to the coordination mechanisms of supply and demand of ecological protection of cultivated land. At the supply side, the research has been concerned with the coupling relationship between certain economic and social factors, and the supply of ecological function of cultivated lands at different spatial scales [12,13,14,15], but there is a lack of consideration of the multiple demands and preferences of stakeholders [16,17]. Studies in recent years have gradually shifted toward the demand side. Some scholars have used the concepts of willingness and preference to define demand, to analyze the public’s cognition and demand behavior of ecosystem services [1,18]. Farmers’, citizens’ and other stakeholders’ cognition of cultivated land functions and willingness to participate in protecting cultivated land have been popular topics [19]. Some scholars have tried, in a qualitative way, to analyze the mismatch between the supply of goods/services provided by multifunctional agriculture and the social demand. Some scholars have employed land-use survey data, expert interviews, and constructed a matrix to analyze the matching of supply and demand of different land-use service functions [20,21]. However, the research, from the perspective of human psychology, is still weak with respect to the behavior of supply and demand of ecological protection of cultivated land. The ecological protection of cultivated land is a process of interaction between humans and nature [22]. As the direct user and manager of cultivated land, farmers’ supply behavior directly determines the effectiveness of the ecological protection of cultivated land. In addition, public demand for the ecological function of cultivated land directly affects its manifestation, which is very important to improve ecological protection of cultivated land [23,24]. In this article, we tried to answer the following questions: What is the public’s cognition of the ecological function of cultivated land? Are there mismatches between the willingness and behavior, as well as suppliers and demanders of ecological protection of cultivated land?

The structure of this paper is as follows: this section introduces the background, literature review and the research purpose; Section 2 introduces the theoretical basis, hypothesis and the model of our research; Section 3 is the scale design and data collection; Section 4 reports the research results. The final section is the discussion and conclusion.

## 2. Theoretical Basis and Research Hypothesis

Stern’s Value-Belief Norm (VBN) Theory [25], Ajzen’s Theory of Planned Behaviour (TPB) [26], and the New Paradigm scale (NEP) [27] were introduced into our study to analyze suppliers’ and demanders’ cognition, willingness and behavior with respect to the ecological protection of cultivated land.

The supply of the ecological functions of cultivated land can change the utilization pattern of cultivated land [28], and farmers act as suppliers in the process of the ecological protection of cultivated land. Meanwhile, farmers and citizens are the demanders in the process of the ecological protection of cultivated land, since they require cultivated land in order to provide agri-food, fibers and agri-services [1]. In particular, farmers have “dual roles” as suppliers and demanders in the process of ecological protection of cultivated land. Individuals are usually regarded as making rational decisions based on an assessment that revenue is equal to the benefits minus the costs, but the revenue is not the only driver of human behavior. If an individual believes that he/she has a moral obligation to protect himself/herself, other people or the whole ecosystem, he/she will adopt a pro-environment attitude [25]. Most stakeholders, especially farmers as suppliers and demanders, make a trade-off in the process of ecological protection of cultivated land.

**Figure 1:**
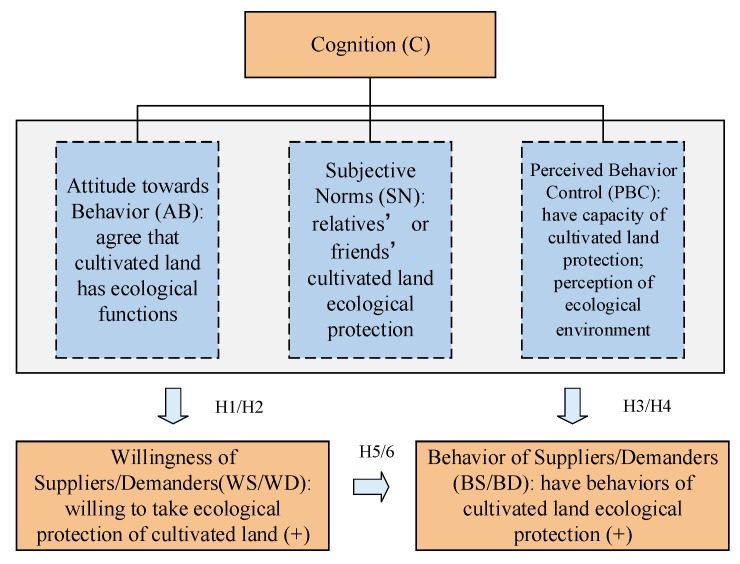
The theoretical framework.

The theory of planned behavior (TPB) based on the theory of Multiattribute Attitude (TMA) and the theory of Reasoned Action (TRA) was expanded to explain and to predict human behavior [26]. This theory is widely used in psychology [29], education [30], health [31], the environment [32], and tourism [33]. It has been proved in practice that the TPB can significantly improve the prediction and explanation of individual behavior [34]. Individual cognition is a key to the ecological protection of cultivated land [35]. As shown in Figure 1, cognition consists of three factors: attitude toward behavior, subjective norms, and perceived behavior control.

Attitude toward behavior refers to the understanding and emotional bias towards the ecological function of cultivated land, is the motive factor of behavior [26]. That is, if the suppliers/demanders can recognize that cultivated land has ecological functions, their willingness and behavior with respect to ecological protection of cultivated land will be correspondingly enhanced. 

Subjective norms refer to the social pressure an individual feels when making a decision as to whether to carry out a specific behavior or not [26]. This means that the individual’s decision-making with respect to the ecological protection of cultivated land is affected by his/her family, relatives and friends. The stronger a supplier’s/demander’s subjective norms, the stronger his/her willingness and behavior will be.

Perceived behavior control is the capacity for the judgment in resources and conditions [26] that an individual can control when he/she has the ability to perform his/her behavior. A higher level of perceived behavior control leads to a stronger willingness and behavior with respect to protecting the ecological environment of cultivated land.

Hence, this paper proposes the following hypotheses.

**Hypothesis 1** **(H1).** *Supplier cognition significantly positively affects willingness with respect to the ecological protection of cultivated land.*

**Hypothesis 2** **(H2).** *Demander cognition significantly positively affects willingness with respect to the ecological protection of cultivated land.*

**Hypothesis 3** **(H3).** *Supplier cognition significantly positively affects behavior with respect to the ecological protection of cultivated land.*

**Hypothesis 4** **(H4).** *Demander cognition significantly positively affects behavior with respect to the ecological protection of cultivated land.*

According to the TPB, supplier/demander behavior comprises consciously induced actions rather than unconscious spontaneous actions [26]. So the willingness to improve the ecological protection of cultivated land will have a positive impact on behavior with respect to the ecological protection of cultivated land. Accordingly, another hypothesis can be stated.

**Hypothesis 5** **(H5).** *Supplier willingness with respect to the ecological protection of cultivated land has a significant positive impact on behavior with respect to the ecological protection of cultivated land.*

**Hypothesis 6** **(H6).** *Demander willingness with respect to the ecological protection of cultivated land has a significant positive impact on behavior with respect to the ecological protection of cultivated land.*

## 3. Material and Methods

### 3.1. Study Area, Participants and Data Sampling

The data in this study comes from the field survey in Caidian District, China in December 2017. Caidian District is located in the southwest of Wuhan City, Hubei Province, China. It is a suburb of Wuhan, and has great diversity in agriculture, industry and tourism. The largest production base in China of lotus root at the district/county level and the Wuhan Economic and Technological Development Zone are both in Caidian District. However, with the development of urbanization, the cultivated land area in Caidian District has gradually decreased. From 2000 to 2015, the cultivated land area decreased from 275,600 hm^2^ to 241,300 hm^2^, and the decline rate in 15 years was 12.45%. The per capita cultivated land area decreased from 0.0291 hm^2^ to 0.0250 hm^2^, which is significantly lower than the warning line of cultivated land area per capita (0.053 ha) put forward by Food and Agriculture Organization of United Nations (FAO) [36]. Due to the improper use of pesticides and chemical fertilizers in the production process, the ecological environment of the cultivated land has been polluted, and the quality of the cultivated land has been degraded [37]. The serious contradiction between human and land makes the ecological protection of cultivated land urgent in Caidian District.

**Figure 2:**
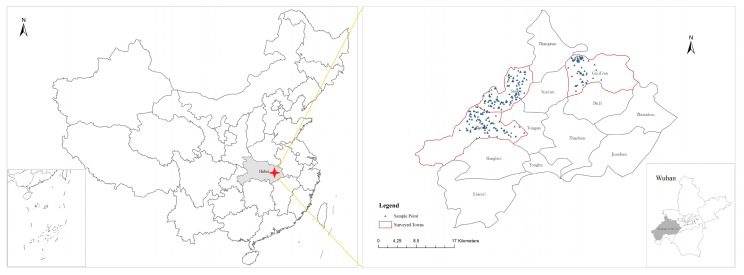
Study area and spatial distribution of sample.

Considering the local social economy, ecological environment and cultivated land resources, we selected three subdistrict (towns)—Caidian (town), Suohe and Zhurushan—as the typical research area (Figure 2). Caidian (town) is close to the Development Zone, with the advantages of a good location and good industrial development. Suohe is rich in natural resources and landscape, and its two villages are listed as the pilot villages of beautiful villages in 2018 in Hubei Province. The area of cultivated land in the street of Zhurushan is the greatest in the whole area, and is mainly used for developing agriculture. To ensure the accuracy and integrity of the survey data, the principle of stratified sampling and random sampling was adopted in the survey. We conducted a pair of face-to-face survey interviews. The basic characteristics of respondents are shown in Table 1. Compared with the statistical data for the Wuhan suburb, the research area is typical, and can be regarded as being representative to a certain extent.

The questionnaire covers the basic information of the respondents (including suppliers/demanders) and their families, and their cognition, willingness and behavior with respect to the ecological protection of cultivated land. To ensure that the respondents have a full understanding of the scale, we did the survey based on the following survey procedure: at first, the terminology related to the ecological function of cultivated land in the scale was explained; second, some pictures of the ecological function of cultivated land were shown to the respondents.

### 3.2. Measurement Instrument

In this study, we designed a five-point Likert scale regarding the suppliers’ and demanders’ cognition, willingness and behavior with respect to ecological protection of cultivated land, with responses ranging from ‘strongly disagree’ (coded 1) to ‘strongly agree’ (coded 5) (Table 2). 

Cognition has three factors: attitude toward behavior, subjective norms, and perceived behavior control. In terms of attitude toward behavior, with reference to relevant research [1,4], combined with the local situation, we considered three functions: water conservation, air purification, and biodiversity protection, and set questions AB1-AB3 accordingly (Table 2) in terms of attitude toward behavior. The “local society”, dominated by China’s consanguinity and geography, cuases the public’s ecological protection of cultivated land behavior to be affected by group pressure [38]. Therefore, it is necessary to select relatives and residents in the same village or neighborhood of the community. For subjective norms, we set questions SN1-SN2 on the degree of participation in the ecological protection of cultivated land. For perceived behavior control, we set questions PBC1-PBC5, considering the individual’s own ability (time, money, physical quality and other abilities), the perception of the cultivated land ecological environment quality, and the public’s degree of difficulty in obtaining the information of ecological protection of cultivated land. In particular, we set the following question for suppliers: “Do you perceive the decrease in soil fertility?”

In terms of willingness and behavior with respect to the ecological protection of cultivated land, we set different questions for suppliers and demanders, respectively. From the perspective of the suppliers, the ecological protection of cultivated land can be regarded as “natural friendly cultivation”, focusing on the characteristics and problems of the ecological environment and the sensitivity of cultivated land [39,40]. Therefore, in this study, we set five questions to investigate willingness (WS1-WS5) and behavior (BS1-BS5) with respect to the ecological protection of cultivated land separately: reducing or not using chemical fertilizer, pesticide and plastic film mulch, straw recycling, and cultivated land fallow. In addition to the production functions of cultivated land for providing agricultural products, demanders need the ecological function of cultivated land to maintain an ideal living environment and to keep food/services from pollution [24]. Therefore, based on the conditional value evaluation method, it is assumed that the ecological function of cultivated land is a kind of commodity, and that there is a perfect market transaction, thus expressing willingness and behavior in terms of willingness to pay or willingness to be compensated [41]. Combined with the classification of the ecological function in the study area, from the perspective of demanders, we set four variables to measure the willingness (WD1-WD4), and three variables to measure the behavior (BD1-BD3) in demanders’ ecological protection of cultivated land.

### 3.3. Reliability and Validity Analysis

To ensure the quality of the survey data and the correctness of the observation variables [42], SPSS 22.0 software was used to test the reliability and validity of the sample data.

Reliability refers to the degree of consistency or stability of the sample measurement results. In this study, Cronbach’s α coefficient was used as the measurement index of reliability test for sample data. The prerequisite for the questionnaire to meet the reliability requirements was that the reliability coefficient of the total scale be above 0.8, and the reliability coefficient of the stratified surface be above 0.5 [43]. The results show that the Cronbach’s α coefficients of the total scale in the supplier group and demander group were 0.975 and 0.970, respectively, and the Cronbach’s α coefficients of the stratified scale were all above 0.8, which shows that the scale has high reliability, reasonable design, and an applicable initial hypothesis path (Table 3).

Validity refers to whether the applied measurement tool can correctly reflect the accuracy of measurement, mainly with respect to content validity and structure validity [44]. Based on the theories and the existing literature, we designed questionnaires, did a trial survey, and modified it to ensure the content validity of the scale. In this study, the Kaiser Meyer Olkin (KMO) measure and Bartlett’s spherical test were performed to determine the structure validity of the scale. The results show that the KMO of the total scale and the stratified surface scale were both higher than the common standard value of 0.7, and the significance level of Bartlett sphere test was 0.000 < 0.01, indicating that the structural validity of all variables is good and suitable for factor analysis [44].

### 3.4. Models

**Figure 3:**
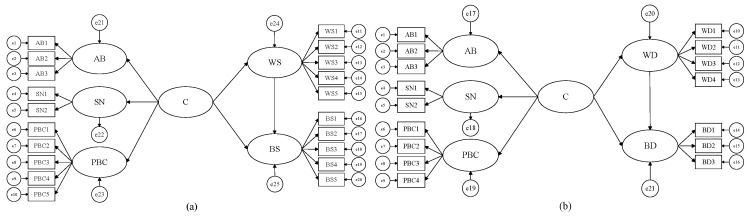
Conceptual model of structural equation of ecological protection of cultivated land on the suppliers’ side (**a**) and on the demanders’ side (**b**).

The structural equation model (SEM), a statistical method based on a covariance structure, was employed to observe the relationship between variables. Based on the Theory of Planned Behavior, in the traditional SEM, we added the variable “Cognition (C)”, composed of the three factors attitude toward behavior, subjective norms and perceived behavior control, and constructed a second-order structural equation model (Figure 3) to explore the ecological protection of cultivated land mechanism of cognition, willingness and behavior of suppliers and demanders.

## 4. Results

### 4.1. Public’s Cognition, Willingness and Behavior

**Figure 4:**
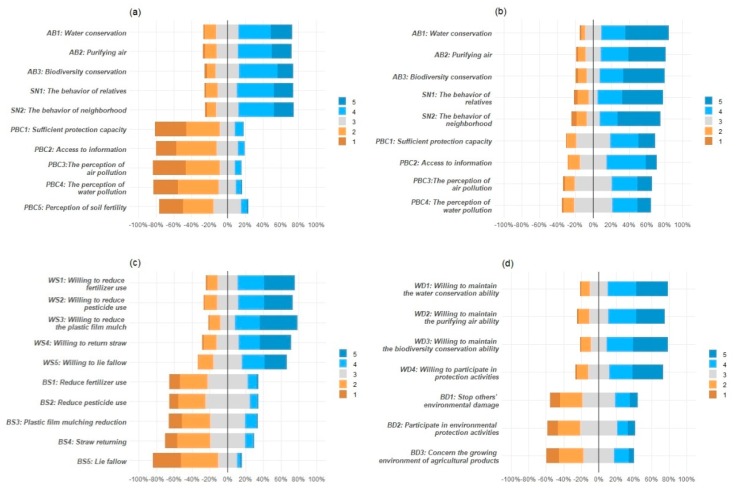
Public cognition, willingness and behavior with respect to the ecological protection of cultivated land. (**a**) Farmers’ cognition; (**b**) Citizens’ cognition; (**c**) Suppliers’ (Farmers’) willingness and behavior; (**d**) Demanders’ (Farmers’ and Citizens’) willingness and behavior.

In terms of cognition (Figure 4a,b), more than 60% of farmers agree that cultivated land can conserve water, purify air and protect biodiversity, and more than 70% of citizens agree with these views. More than 65% of the respondents believe that their family members’ and neighbors’ behavior with respect to the ecological protection of cultivated land would have a positive impact on their own willingness and behavior. The proportion of citizens who agree with these viewpoints is slightly higher than the farmers. It is shown that the public’s attitude toward behavior and subjective norms are at a high level, and the cognition of citizens in these two dimensions is slightly higher than that of farmers. However, for the perceived behavior control, there is a big difference between farmers and citizens. More than 60% of citizens think that they have enough capacity and access to information on how to protect the ecological environment of cultivated land, and they can perceive the deterioration of the ecological environment, while less than 20% of farmers do so. The cognition of citizens is generally higher than that of farmers, which is especially true of perceived behavior control. Farmers’ capacity for the ecological protection of cultivated land is obviously insufficient, access to information on how to ecologically protect cultivated land is limited, and awareness of ecological environment deterioration is lacking. We found that there is a “mismatch” between the farmers and the citizens with respect to cognition.

Concerning willingness (Figure 4b) and behavior (Figure 4c), more than 80% of suppliers and demanders are willing to protect the ecological environment of cultivated land, but behavior is far lower than willingness. Although nearly 80% of suppliers and demanders are willing to protect the cultivated land environment, in actual agricultural production, less than 35% of suppliers adopt “naturally friendly farming” such as using less pesticide and chemical fertilizer and reducing the use of plastic film. In addition, only about 40% of those who participate in environmental protection activities of cultivated land prevent others from damaging the ecological environment of cultivated land and pay attention to the ecological environment of the producing area when purchasing agricultural products. In terms of willingness and behavior with respect to the ecological protection of cultivated land, there is not only a “mismatch” between the suppliers and the demanders, but also a more serious “mismatch” between willingness and behavior.

### 4.2. Interactive Mechanism of Cognition, Willingness and Behavior with Respect to the Ecological Protection of Cultivated Land

In this section, we used SEM to test the hypothesis, exploring the mechanisms for the ecological protection of cultivated land from the perspectives of cognition, willingness and behavior. Amos 17.0 software (SPSS Inc., Chicago, IL, USA) was employed.

**Figure 5:**
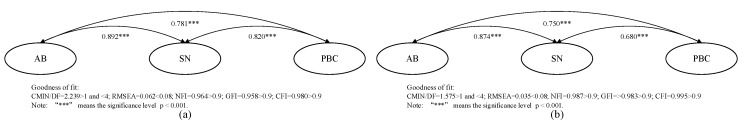
The interactions between attitude and behavior, subjective norms and perceived behavior control.

Most of the existing literature focuses on cognition as a whole, ignoring the possible interaction among various dimensions in the cognitive structure [45]. Therefore, firstly, a Multiple Factor Skew model is used to verify the cognitive measurement model of the ecological function of cultivated land. The results of the model fitness test show that that all indicators are within the acceptable range (Figure 4), which shows that the fitness of the overall model and data is good. The standardized compliance factors of attitude toward behavior, subjective norms and perceived behavior control are all greater than 0.7 and all reach the significance level of *p* < 0.001. The combined reliability index (CR) is more than 0.8, and the aggregate validity index (AVE) is more than 0.5, which indicates that the model has a good internal fit. There are significant interactions among the three factors in the supplier group and in demander group (Figure 5), which shows that there are higher-order common factors, and that the second-order Structural Equation Model is suitable for further verifying the mechanism of cognition, willingness and behavior with respect to the ecological protection of cultivated land.

**Figure 6:**
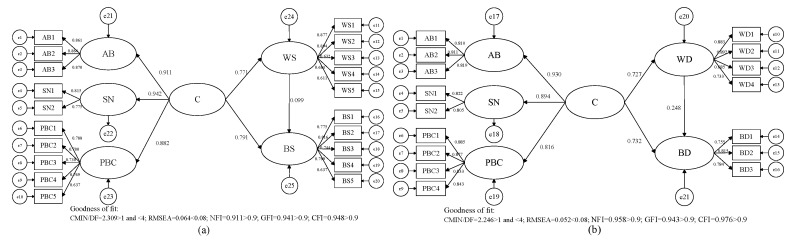
Route analysis of public cognition, willingness and behavior with respect to ecological protection of cultivated land on the supplier side (**a**) and on the demander side (**b**).

Then, the maximum likelihood method is used to estimate the path parameters of the hypothetical model, and we obtained the fitting results of the ecological protection of cultivated land models in the supplier group and the demander group (Figure 6). The overall fitness evaluation of the models shows that the fitting indexes all meet the fitting standard.

Observing the path coefficients in the models, it can be found that hypotheses H1 and H3 on the supplier side pass the test at the level of *p* < 0.001 (***), and the coefficient symbols meet the expectations, but hypothesis H5 fails the test. The hypotheses H2, H4 and H6 on the demander side pass the test at a significance level of *p* < 0.001 (***), and the coefficient symbols meet the expectations (Table 4).

To further explore the relationships among potential variables, we calculated the direct effect, indirect effect and total effect of each potential variable. Direct effect refers to the direct influence of the cause variable on the result variable, which is measured by the path coefficient of the two potential variables. Indirect effect refers to the indirect effect of the cause variable on the result variable when there are intermediate variables in the model, which is expressed by the product of the path coefficients in the influence interval. Total effect refers to the total effect from cause variable to result variable, which is the sum of direct effect and indirect effect. The results are shown in Table 5. In addition, it is shown that the willingness and behavior of the suppliers and demanders to protect cultivated land ecological environment are positively affected to a great extent by the cognition of the ecological function of cultivated land. In particular, compared with the suppliers, there is a direct effect of 0.326 between the willingness and the behavior of the demanders, so there is an indirect effect of 0.234 between cognition and behavior, and the total effect between cognition and willingness is 0.867. This means that the behavior of demanders also depends on willingness. Willingness plays an intermediary variable role, that is, cognition directly affects the behavior, and indirectly affects behavior through willingness. There are some differences in the mechanisms of cognition, willingness and behavior between suppliers and demanders.

## 5. Discussion

In our study, to explore the public’s cognition, willingness and behavior with respect to the ecological protection of cultivated land, we designed a five-point Likert scale from the perspective of suppliers (farmers) and demanders (farmers and citizens), and obtained a lot of interesting and meaningful findings:

Firstly, in terms of cognition factor, the attitudes toward the behavior and subjective norms for farmers and citizens are all at a high level. Most of the farmers and citizens recognize the functions of cultivated land functions with respect to purifying air, conserving water and protecting biodiversity. Behavior is affected by their family and neighborhoods. However, perceived behavior control is at a relatively low level. The perception of cultivated land ecological environment, the capacity and access to information are relatively low, especially in the farmers’ group. Compared with citizens, farmers have less convenient access to information, and they have lower recognition of the ecological functions of cultivated land. Farmers’ perception of air pollution and water pollution is lower than citizens. Farmers are the direct managers of cultivated land, but they do not perceive the deterioration of the ecological environment of cultivated land, and they have lower capacities, and lack access to information on cultivated land protection, which could be a huge obstacle for the future ecological protection of cultivated land.

Secondly, behavior is lower than willingness, and there was a mismatch with willingness in both the supplier and demander groups. In the demander group, farmers and citizens have strong willingness to ecologically protect the cultivated land, but the behavior is greatly reduced when considering the impact of their own economic conditions and external environmental factors. In the supplier/farmer group, under their current cognition, it is difficult for farmers to predict whether their income will be reduced due to engaging in the ecological protection of cultivated land, so it is also difficult to achieve “friendly cultivation of cultivated land”. The “mismatch” in the supplier group is more serious than in the demander group. On the one hand, the citizens as demanders are far away from the location of the cultivated land, and they lack communication (channels) directly with the suppliers/farmer, so it is difficult for them to engage in behavior. On the other hand, because farmers have dual roles as both suppliers and demanders, there is a trade-off between the ecological protection of cultivated land and economic interests in their decision-making process with respect to supply and demand in terms of willingness, and especially in terms of behavior. If the farmers make a decision based on economic interests, it would be extremely unfavorable to the sustainable ecological protection of cultivated land.

Thirdly, there are differences in the mechanisms of cognition, willingness and behavior both in the supplier group and the demander group. On the supplier side, subjective norms have the highest impact, followed by attitude toward behavior, and perceived behavior control has the lowest impact on willingness and behavior in the ecological protection of cultivated land. Because of the blood and geographical relationships in China, suppliers are more likely to succumb to group pressure, and the ecological protection of cultivated land is usually collective/group oriented. However, on the demander side, attitude toward behavior has the highest level, while subjective norms and perceived behavior control have relatively low levels. Demanders’ willingness and behavior are more susceptible to their own attitudes. Therefore, we should increase the public’s cognition/awareness of the ecological function of cultivated lands, and improve the incentive–restrictive mechanism to enhance the public’s willingness and behaviors with respect to the ecological protection of cultivated land.

In particular, we found that the willingness on the demanders’ side plays an intermediary effect, while the intermediary effect of willingness on the suppliers’ side was not verified. As for citizens, the impact of cognition on willingness is in line with the Theory of Planned Behavior. However, farmers, as suppliers and demanders, have differences in cognition, willingness and behavior. There is a self-contradiction. Farmers need to rely on planting and selling food, vegetables and other crops to gain economic benefits for their livelihood [46]. Using chemical fertilizers, pesticide and plastic film is the most common way to reduce pests and increase yield. However, at this stage, the farmers generally have the behavior of nonstandard fertilization and application of pesticide [46,47], and the popularity of ecological protection methods such as returning of straw and the use of fallow periods are not enough. At the same time, farmers are also demanders who consume the agricultural products they produce, and which they buy from the market [48]. Like the citizens as demanders, farmers as demanders also pay attention to food safety, but there is a phenomenon in the countryside: the farmers sell the crops, fruits and vegetables with more pesticides, leaving the part of the agricultural products with less for their own consumption. This reflects the dilemma of the farmers’ own interests and social interests, which can contribute to explaining the mismatch between their roles as suppliers and as demanders, and the mismatch between willingness and behavior. In the future, we should offer ecological planting technology training, and improve farmers’ knowledge of the ecological protection of cultivated land. More importantly, we should perfect agri-ecological goods/service market mechanism via eco-indication, and encourage some new agricultural forms like community-supported agriculture (CSA), an effective bridge of suppliers and demanders.

Our current research can provide some reference for the research area and beyond. However, there are still some deficiencies in the research. Firstly, in our study, we chose three towns with traditional agriculture, industry and ecological agriculture as the study area, but we did not do it under different scenarios. The question of interactive mechanism of cognition, willingness and behavior under different scenarios would be very interesting for the future research. Secondly, farmers with dual roles as demanders and as suppliers are an important research object, especially in terms of cognition, willingness, behavior, and relationships, which should be more deeply analyzed in a more systematic way.

## 6. Conclusions

Employing a sample of 461 farmers and citizens from Caidian District, Wuhan China, and based on a five-point Likert scale, in this article we initially analyzed suppliers’ and demanders’ cognition, willingness and behavior with respect to the ecological protection of cultivated land at the micro-level. We found that there were mismatches in willingness and behavior with respect to the ecological protection of cultivated land within the supplier group as well as within the demander group, and also between the two groups. We also revealed the difference between the supplier group and the demander group in terms of interplay mechanism of cognition, willingness and behavior. We discussed the reasons behind these results, and put forward some suggestions for the ecological protection of cultivated land. In the future, we should pay more attention to the improvement of the cognition of the ecological function of cultivated lands and the ecological protection skills of farmers, who are both suppliers and demanders. Only when farmers have the capacity to realize the coordination of economic interests and social interests, that is, the coordination of their own supply and demand, can they achieve coordination of their willingness and behavior. In this way, the coordination of supply and demand of farmers and citizens can be realized, so as to ensure the orderly progress of ecological protection of cultivated land, the manifestation of the ecological function of cultivated land, and the achievement of sustainable use of cultivated land.

## Figures and Tables

**Table 1:** Basic characteristics of interviewees.

Demographic Characteristics	Farmers	Citizens	Demographic Characteristics	Farmers	Citizens
	*n* (%)	*n* (%)		*n* (%)	*n* (%)
Gender			Education		
Male	162 (50.15)	69 (50.00)	Elementary school education or even lower	137 (42.41)	40 (28.99)
Female	161 (49.85)	69 (50.00)	Junior middle school	100 (30.96)	41 (29.71)
Age			Senior high school or technical secondary school	60 (18.58)	33 (23.91)
Under 26 years old	5 (1.55)	13 (9.42)	Senior high school or above	26 (8.05)	24 (17.39)
26–35	30 (9.29)	18 (10.71)	Family income		
36–45	47 (14.55)	18 (10.71)	Under 20000 yuan	89 (27.55)	31 (22.46)
46–55	79 (24.46)	32 (23.19)	20000-50000 yuan	168 (52.01)	16 (11.60)
56–65	87 (26.94)	24 (14.50)	Over 50000 yuan	67 (20.74)	91 (65.94)
66–75	53 (16.41)	27 (19.57)			
Over 75 years old	22 (6.81)	6 (6.52)	Total	323	138

**Table 2:** Scale of public cognition, willingness and behavior in ecological protection of cultivated land.

Code	Item	M (SD)	
Cognitive (C)		Farmers	Citizens
AB1	In my opinion, cultivated land has the function of water conservation.	3.70 (1.01)	4.18 (0.98)
AB2	In my opinion, cultivated land has the function of purifying air.	3.66 (1.04)	4.01 (1.05)
AB3	In my opinion, cultivated land has the function of biodiversity conservation.	3.62 (0.99)	4.14 (1.12)
SN1	My family or relatives have contributed to the ecological protection of cultivated land.	3.69 (1.01)	3.98 (1.21)
SN2	My friends or neighborhood have contributed to the ecological protection of cultivated land.	3.71 (0.99)	3.94 (1.27)
PBC1	I have enough capacity to protect the cultivated land ecological environment.	2.03 (0.99)	3.57 (0.94)
PBC2	I can access enough information about the ecological protection of cultivated land.	2.17 (0.88)	3.54 (0.90)
PBC3	I have perceived the increasing air pollution.	1.97 (0.94)	3.47 (0.97)
PBC4	I have perceived the increasing water pollution.	2.07 (1.91)	3.42 (0.96)
PBC5	I have perceived the decrease of soil fertility. (only for farmers)	2.22 (0.96)	——
Willingness of suppliers (WS)			
WS1	I’m willing to reduce the chemical fertilizer used in cultivating.	3.85 (1.06)	
WS2	I’m willing to reduce the pesticides used in cultivating.	3.76 (1.09)	
WS3	I’m willing to reduce the plastic film mulch used in cultivating.	3.98 (1.08)	
WS4	I’m willing to return to straw in cultivating.	3.76 (1.13)	
WS5	I’m willing to lie fallow in cultivating.	3.58 (1.05)	
Willingness of demanders (WD)			
WD1	I’m willing to maintain the water conservation function of cultivated land.	3.92 (1.03)	
WD2	I’m willing to maintain the purifying air function of cultivated land.	3.82 (1.07)	
WD3	I’m willing to maintain the biodiversity conservation function of cultivated land.	3.97 (1.05)	
WD4	I’m willing to participate in ecological protection of cultivated land activities.	3.81 (1.11)	
Behavior of suppliers (BS)			
BS1	I have reduced the chemical fertilizer using in cultivating.	2.59 (0.89)	
BS2	I have reduced the pesticides using in cultivating.	2.60 (0.82)	
BS3	I have reduced the plastic film mulsh using in cultivating.	2.54 (0.94)	
BS4	I have returned straw in cultivating.	2.47 (0.88)	
BS5	I have laid fallow in cultivating.	2.01 (0.89)	
Behavior of demanders (BD)			
BD1	I often stop others’s damage to the ecological environment of cultivated land.	2.88 (1.11)	
BD2	I often participate in activities towards the ecological protection of cultivated land.	2.80 (1.07)	
BD3	I often consider the growing environment of agricultural products when I buy them.	2.73 (1.09)	

Note: M = mean, SD = standard deviation; AB = attitude toward behavior, SN = subjective norms, PBC= perceived behavior control.

**Table 3:** Reliability and validity test results of the scale.

Group	Item	Cronbach’ s α	KMO	Bartlett’s Spherical Test		
				$\chi^{2}$	df	Sig.
The scale of supplier group	Total scale	0.951	0.954	4389.257	210	0.000
	AB	0.899	0.751	592.893	3	0.000
	SN	0.774	0.709	285.121	1	0.000
	PBC	0.854	0.851	680.311	10	0.000
	WS	0.857	0.798	806.653	10	0.000
	BS	0.881	0.889	908.606	15	0.000
The scale of demander group	Total scale	0.945	0.949	5193.047	120	0.000
	AB	0.855	0.733	609.238	3	0.000
	SN	0.796	0.709	285.121	1	0.000
	PBC	0.918	0.849	1318.677	6	0.000
	WD	0.875	0.781	1032.477	6	0.000
	BD	0.826	0.716	512.365	3	0.000

**Table 4:** Regression results of variables of SEM.

Group	Path	Estimate	C.R	S.E.	Result
Suppliers	AB→C	0.911 ***	——	——	Accept
	SN→C	0.942 ***	0.067	14.780	Accept
	PBC→C	0.882 ***	0.061	12.473	Accept
	C→WS	0.771 ***	0.068	9.974	Accept
	C→BS	0.791 ***	0.082	8.611	Accept
	WS→BS	0.099	0.077	1.309	Reject
Demanders	AB→C	0.930 ***	——	——	Accept
	SN→C	0.894 ***	0.063	15.776	Accept
	PBC→C	0.816 ***	0.063	15.357	Accept
	C→WD	0.727 ***	0.060	12.337	Accept
	C→BD	0.732 ***	0.073	10.809	Accept
	WD→BD	0.248 ***	0.059	4.435	Accept

*** means the significance level *p* < 0.001

**Table 5:** The standardized results of direct effects, indirect effects and total effects among the potential variables in the model.

Variable	C	W(WS/WD)			B (BS/BD)		
	DE	DE	IE	TE	DE	IE	TE
Suppliers							
AB	0.908	0.000	0.702	0.702	0.000	0.732	0.732
SN	0.940	0.000	0.727	0.727	0.000	0.758	0.758
PBC	0.888	0.000	0.686	0.686	0.000	0.716	0.716
C	0.000	0.773	0.000	0.773	0.806	0.000	0.806
WS	0.000	0.000	0.000	0.000	0.000	0.000	0.000
Demanders							
AB	0.945	0.000	0.678	0.678	0.000	0.598	0.598
SV	0.924	0.000	0.663	0.663	0.000	0.585	0.585
PBC	0.792	0.000	0.568	0.568	0.000	0.501	0.501
WD	0.000	0.717	0.000	0.717	0.633	0.234	0.867
BD	0.000	0.000	0.000	0.000	0.326	0.000	0.326

Note. W = Willingness, B = Behavior, DE = Direct Effect, IE = Indirect Effect, TE = Total Effect.

## References

[1] Wolff S., Schulp C.J.E., Kastner T., Verburg P.H. (2017). Quantifying Spatial Variation in Ecosystem Services Demand: A Global Mapping Approach. Ecol. Econ..

[2] De Groot R. (2006). Function-analysis and valuation as a tool to assess land use conflicts in planning for sustainable, multi-functional landscapes. Landsc. Urban Plan..

[3] Wilson G.A. (2009). The spatiality of multifunctional agriculture: A human geography perspective. Geoforum.

[4] Van Huylenbroeck G., Vandermeulen V., Mettepenningen E., Verspecht A. (2007). Multifunctionality of Agriculture: A Review of Definitions, Evidence and Instruments. Living Rev. Landsc. Res..

[5] Yang B., Shang J., Yu F. (2019). Difficulty, problems and countermeasures of agricultural non-point sources pollution control in China. Chin. J. Eco Agric..

[6] Liu D., Gong Q., Yang W. (2018). The Evolution of Farmland Protection Policy and Optimization Path from 1978 to 2018. Chin. Rural Econ..

[7] Špur N., Škornik S., Šorgo A. (2019). Influence of attitudinal dimensions on children’s interest in preserving extensive grasslands. J. Rural Stud..

[8] Limitcd P. (1995). A Structural Model of Environmental Behaviour Attitudes. J. Environ. Psychol..

[9] Cordano M., Welcomer S., Scherer R., Pradenas L., Parada V. (2010). Understanding cultural differences in the antecedents of pro-environmental behavior: A comparative analysis of business students in the United States and Chile. J. Environ. Educ..

[10] Bennett N.J., Roth R., Klain S.C., Chan K., Christie P., Clark D.A., Cullman G., Curran D., Durbin T.J., Epstein G. (2017). Conservation social science: Understanding and integrating human dimensions to improve conservation. Biol. Conserv..

[11] Zasada I. (2011). Multifunctional peri-urban agriculture-A review of societal demands and the provision of goods and services by farming. Land Use Policy.

[12] Schröter M., Barton D.N., Remme R.P., Hein L. (2014). Accounting for capacity and flow of ecosystem services: A conceptual model and a case study for Telemark, Norway. Ecol. Indic..

[13] Tallis H., Kareiva P., Marvier M., Chang A., Mwinyi A.H. (2008). Practical Conservation and Economic Development. Proc. Natl. Acad. Sci. USA.

[14] García-Nieto A.P., Geijzendorffer I.R., Baró F., Roche P.K., Bondeau A., Cramer W. (2018). Impacts of urbanization around Mediterranean cities: Changes in ecosystem service supply. Ecol. Indic..

[15] Zhang Y., Long H., Ge D., Tu S., Qu Y. (2018). Spatio-temporal characteristics and dynamic mechanism of farmland functions evolution in the Huang-Huai-Hai Plain. Dili Xuebao Acta Geogr. Sin..

[16] Hu W., Wei A., Zhao Z., Zhang A. (2017). Literature Review on Mismatch of Demand and Supply, and Synergies of Multifunctional Agricultural Land. China Land Sci..

[17] Bai Y., Wang M., Li H., Huang S.F., Alatalo J.M. (2017). Ecosystem service supply and demand: Theory and management application. Shengtai Xuebao Acta Ecol. Sin..

[18] Casado-Arzuaga I., Madariaga I., Onaindia M. (2013). Perception, demand and user contribution to ecosystem services inthe Bilbao Metropolitan Greenbelt. J. Environ. Manag..

[19] Lee C., Liao L., Chen Y., Wang Y., Lan I. (2009). Farmland Functions and Use Types Option under Multfunctional Agricultural Regime. J. TWN Land Res..

[20] Zasada I., Fertner C., Piorr A., Nielsen T.S. (2011). Peri-urbanisation and multifunctional adaptation of agriculture around Copenhagen. Geogr. Tidsskr..

[21] Burkhard B., Kroll F., Nedkov S., Müller F. (2012). Mapping ecosystem service supply, demand and budgets. Ecol. Indic..

[22] Costanza R., Fisher B., Ali S., Beer C., Bond L., Boumans R., Danigelis N.L., Dickinson J., Elliott C., Farley J. (2007). Quality of life: An approach integrating opportunities, human needs, and subjective well-being. Ecol. Econ..

[23] Lefroy R.D.B., Bechstedt H.D., Rais M. (2000). Indicators for sustainable land management based on farmer surveys in Vietnam, Indonesia, and Thailand. Agric. Ecosyst. Environ..

[24] Wolff S., Schulp C.J.E., Verburg P.H. (2015). Mapping ecosystem services demand: A review of current research and future perspectives. Ecol. Indic..

[25] Stern P.C., Dietz T., Abel T., Guagnano G.A., Kalof L. (1999). A value-belief-norm theory of support for social movements: The case of environmentalism. Hum. Ecol. Rev..

[26] Ajzen I. (1991). The theory of planned behavior. Organ. Behav. Hum. Decis. Process..

[27] Dunlap R.E., Van Liere K.D., Mertig A.G., Jones R.E. (2000). New Trends in Measuring Environmental Attitudes: Measuring Endorsement of the New Ecological Paradigm: A Revised NEP Scale. J. Soc. Issues.

[28] Kong X., Zhang B., Wen L., Hu Y., Lei M., Yao J., Xin Y. (2018). Theoretical Framework and Research Trends of Cultivated Land Quality based on Elements-Process-Function. China Land Sci..

[29] Fung X.C.C., Pakpour A.H., Wu Y.K., Fan C.W., Lin C.Y., Tsang H.W.H. (2019). Psychosocial Variables Related to Weight-Related Self-Stigma in Physical Activity among Young Adults across Weight Status. Int. J. Environ. Res. Public Health.

[30] Cheon J., Lee S., Crooks S.M., Song J. (2012). An investigation of mobile learning readiness in higher education based on the theory of planned behavior. Comput. Educ..

[31] Gu D., Guo J., Liang C., Lu W., Zhao S., Liu B., Long T. (2019). Social media-based health management systems and sustained health engagement: TPB perspective. Int. J. Environ. Res. Public Health.

[32] Han H., Hsu L.T., Sheu C. (2010). Application of the Theory of Planned Behavior to green hotel choice: Testing the effect of environmental friendly activities. Tour. Manag..

[33] Quintal V.A., Lee J.A., Soutar G.N. (2010). Risk, uncertainty and the theory of planned behavior: A tourism example. Tour. Manag..

[34] Ajzen I., Driver B.L. (1992). Application of the Theory of Planned Behavior to Leisure Choice. J. Leis. Res..

[35] De Leeuw A., Valois P., Ajzen I., Schmidt P. (2015). Using the theory of planned behavior to identify key beliefs underlying pro-environmental behavior in high-school students: Implications for educational interventions. J. Environ. Psychol..

[36] Ran Q., Yue Y., Xie D., Wei C., Ran R. (2007). Calculating the Threshold Value of Per Capita Arable Land Security in China. Res. Sci..

[37] Li S., Gong Q., Yang S. (2019). Analysis of the agricultural economy and agricultural pollution using the decoupling index in Chengdu, China. Int. J. Environ. Res. Public Health.

[38] Schweers Cook K. (2005). Networks, Norms, and Trust: The Social Psychology of Social Capital 2004 Cooley Mead Award Address. Soc. Psychol. Q..

[39] Shi T. (2002). Ecological agriculture in China: Bridging the gap between rhetoric and practice of sustainability. Ecol. Econ..

[40] Ye X.J., Wang Z.Q., Li Q.S. (2002). The ecological agriculture movement in modern China. Agric. Ecosyst. Environ..

[41] Villamagna A.M., Angermeier P.L., Bennett E.M. (2013). Capacity, pressure, demand, and flow: A conceptual framework for analyzing ecosystem service provision and delivery. Ecol. Complex..

[42] Bacon D.R., Sauer P.L., Young M. (1995). Composite Reliability in Structural Equations Modeling. Educ. Psychol. Meas..

[43] Cronbach L.J. (1951). Coefficient alpha and the internal structure of tests. Psychometrika.

[44] Field A. (2005). Discovering Statistics Using IBM SPSS Statistics.

[45] Hu W., Li M., Zhang J., Zhu Q. (2019). Research on farmers’ supply behavior of agricultural land ecological function: Based on ectended theory of planning behavior. Chin. J. Agric. Resour. Reg. Plan..

[46] Wang W., Jin J., He R., Gong H. (2017). Gender differences in pesticide use knowledge, risk awareness and practices in Chinese farmers. Sci. Total Environ..

[47] Jin J., Wang W., He R., Gong H. (2017). Pesticide use and risk perceptions among small-scale farmers in Anqiu County, China. Int. J. Environ. Res. Public Health.

[48] Huang Y., Luo X. (2018). Eating and selling: Analysis on the difference of the application behavior of bio pesticide of rice farmers. Chin. Rural Econ..

